# The therapeutic potential of stem cells

**DOI:** 10.1098/rstb.2009.0149

**Published:** 2010-01-12

**Authors:** Fiona M. Watt, Ryan R. Driskell

**Affiliations:** Wellcome Trust Centre for Stem Cell Research, University of Cambridge, Tennis Court Road, Cambridge CB2 1QR, UK

**Keywords:** adult stem cells, ES cells, iPS cells, cell-based therapies, drug discovery

## Abstract

In recent years, there has been an explosion of interest in stem cells, not just within the scientific and medical communities but also among politicians, religious groups and ethicists. Here, we summarize the different types of stem cells that have been described: their origins in embryonic and adult tissues and their differentiation potential *in vivo* and in culture. We review some current clinical applications of stem cells, highlighting the problems encountered when going from proof-of-principle in the laboratory to widespread clinical practice. While some of the key genetic and epigenetic factors that determine stem cell properties have been identified, there is still much to be learned about how these factors interact. There is a growing realization of the importance of environmental factors in regulating stem cell behaviour and this is being explored by imaging stem cells *in vivo* and recreating artificial niches *in vitro*. New therapies, based on stem cell transplantation or endogenous stem cells, are emerging areas, as is drug discovery based on patient-specific pluripotent cells and cancer stem cells. What makes stem cell research so exciting is its tremendous potential to benefit human health and the opportunities for interdisciplinary research that it presents.

## 1. Introduction: what are stem cells?

The human body comprises over 200 different cell types that are organized into tissues and organs to provide all the functions required for viability and reproduction. Historically, biologists have been interested primarily in the events that occur prior to birth. The second half of the twentieth century was a golden era for developmental biology, since the key regulatory pathways that control specification and morphogenesis of tissues were defined at the molecular level (Arias 2008). The origins of stem cell research lie in a desire to understand how tissues are maintained in adult life, rather than how different cell types arise in the embryo. An interest in adult tissues fell, historically, within the remit of pathologists and thus tended to be considered in the context of disease, particularly cancer.

**Figure 1.**
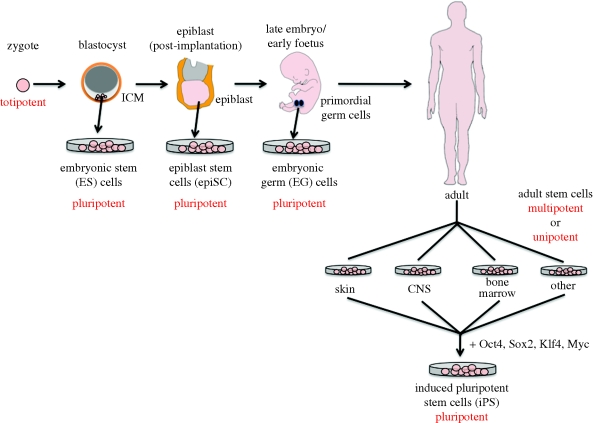
Origin of stem cells. Cells are described as *pluripotent* if they can form all the cell types of the adult organism. If, in addition, they can form the extraembryonic tissues of the embryo, they are described as *totipotent. Multipotent* stem cells have the ability to form all the differentiated cell types of a given tissue. In some cases, a tissue contains only one differentiated lineage and the stem cells that maintain the lineage are described as *unipotent*. Postnatal spermatogonial stem cells, which are unipotent *in vivo* but pluripotent in culture, are not shown (Jaenisch & Young 2008). CNS, central nervous system; ICM, inner cell mass.

It was appreciated long ago that within a given tissue there is cellular heterogeneity: in some tissues, such as the blood, skin and intestinal epithelium, the differentiated cells have a short lifespan and are unable to self-renew. This led to the concept that such tissues are maintained by stem cells, defined as cells with extensive renewal capacity and the ability to generate daughter cells that undergo further differentiation (Lajtha 1979). Such cells generate only the differentiated lineages appropriate for the tissue in which they reside and are thus referred to as multipotent or unipotent (figure 1).

In the early days of stem cell research, a distinction was generally made between three types of tissue: those, such as epidermis, with rapid turnover of differentiated cells; those, such as brain, in which there appeared to be no self-renewal; and those, such as liver, in which cells divided to give two daughter cells that were functionally equivalent (Leblond 1964; Hall & Watt 1989). While it remains true that different adult tissues differ in terms of the proportion of proliferative cells and the nature of the differentiation compartment, in recent years it has become apparent that some tissues that appeared to lack self-renewal ability do indeed contain stem cells (Zhao *et al*. 2008) and others contain a previously unrecognized cellular heterogeneity (Zaret & Grompe 2008). That is not to say that all tissues are maintained by stem cells; for example, in the pancreas, there is evidence against the existence of a distinct stem cell compartment (Dor *et al*. 2004).

One reason why it took so long for stem cells to become a well-established research field is that in the early years too much time and energy were expended in trying to define stem cells and in arguing about whether or not a particular cell was truly a stem cell (Watt 1999). Additional putative characteristics of stem cells, such as rarity, capacity for asymmetric division or tendency to divide infrequently, were incorporated into the definition, so that if a cell did not exhibit these additional properties it tended to be excluded from the stem cell ‘list’. Some researchers still remain anxious about the definitions and try to hedge their bets by describing a cell as a stem/progenitor cell. However, this is not useful. The use of the term progenitor, or transit amplifying, cell should be reserved for a cell that has left the stem cell compartment but still retains the ability to undergo cell division and further differentiation (Potten & Loeffler 2008).

**Figure 2.**
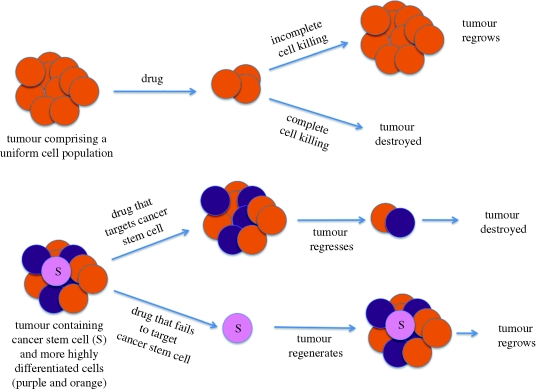
The cancer stem cell hypothesis. The upper tumour is shown as comprising a uniform population of cells, while the lower tumour contains both cancer stem cells and more differentiated cells. Successful or unsuccessful chemotherapy is interpreted according to the behaviour of cells within the tumour.

Looking back at some of the early collections of reviews written as the proceedings of stem cell conferences, one regularly finds articles on the topic of cancer stem cells (McCulloch *et al*. 1988). However, these cells have only recently received widespread attention (Reya *et al*. 2001; Clarke *et al*. 2006; Dick 2008). The concept is very similar to the concept of normal tissue stem cells, namely that cells in tumours are heterogeneous, with only some, the cancer stem cells, or tumour initiating cells, being capable of tumour maintenance or regrowth following chemotherapy. The cancer stem cell concept is important because it suggests new approaches to anti-cancer therapies (figure 2).

As in the case of tissue stem cells, it is important that cancer stem cell research is not sidetracked by arguments about definitions. It is quite likely that in some tumours all the cells are functionally equivalent, and there is no doubt that tumour cells, like normal stem cells, can behave differently under different assay conditions (Quintana *et al*. 2008). The oncogene dogma (Hahn & Weinberg 2002), that tumours arise through step-wise accumulation of oncogenic mutations, does not adequately account for cellular heterogeneity, and markers of stem cells in specific cancers have already been described (Singh *et al*. 2004; Barabé *et al*. 2007; O'Brien *et al*. 2007). While the (rediscovered) cancer stem cell field is currently in its infancy, it is already evident that a cancer stem cell is not necessarily a normal stem cell that has acquired oncogenic mutations. Indeed, there is experimental evidence that cancer initiating cells can be genetically altered progenitor cells (Clarke *et al*. 2006).

In addition to adult tissue stem cells, stem cells can be isolated from pre-implantation mouse and human embryos and maintained in culture as undifferentiated cells (figure 1). Such embryonic stem (ES) cells have the ability to generate all the differentiated cells of the adult and are thus described as being pluripotent (figure 1). Mouse ES cells are derived from the inner cell mass of the blastocyst, and following their discovery in 1981 (Evans & Kaufman 1981; Martin 1981) have been used for gene targeting, revolutionizing the field of mouse genetics. In 1998, it was first reported that stem cells could be derived from human blastocysts (Thomson *et al*. 1998), opening up great opportunities for stem cell-based therapies, but also provoking controversy because the cells are derived from ‘spare’ *in vitro* fertilization embryos that have the potential to produce a human being. It is interesting to note that, just as research on adult tissue stem cells is intimately linked to research on disease states, particularly cancer, the same is true for ES cells. Many years before the development of ES cells, the *in vitro* differentiation of cells derived from teratocarcinomas, known as embryonal carcinoma cells, provided an important model for studying lineage selection (Andrews *et al*. 2005).

Blastocysts are not the only source of pluripotent ES cells (figure 1). Pluripotent epiblast stem cells, known as epiSC, can be derived from the post-implantation epiblast of mouse embryos (Brons *et al*. 2007; Tesar *et al*. 2007). Recent gene expression profiling studies suggest that human ES cells are more similar to epiSC than to mouse ES cells (Tesar *et al*. 2007). Pluripotent stem cells can also be derived from primordial germ cells (EG cells), progenitors of adult gametes, which diverge from the somatic lineage at late embryonic to early foetal development (Kerr *et al*. 2006).

Although in the past the tendency has been to describe ES cells as pluripotent and adult stem cells as having a more restricted range of differentiation options, adult cells can, in some circumstances, produce progeny that differentiate across the three primary germ layers (ectoderm, mesoderm and endoderm). Adult cells can be reprogrammed to a pluripotent state by transfer of the adult nucleus into the cytoplasm of an oocyte (Gurdon *et al*. 1958; Gurdon & Melton 2008) or by fusion with a pluripotent cell (Miller & Ruddle 1976). The most famous example of cloning by transfer of a somatic nucleus into an oocyte is the creation of Dolly the sheep (Wilmut *et al*. 1997). While the process remains inefficient, it has found some unexpected applications, such as cloning endangered species and domestic pets.

A flurry of reports almost 10 years ago suggested that adult cells from many tissues could differentiate into other cell types if placed in a new tissue environment. Such studies are now largely discredited, although there are still some bona fide examples of transdifferentiation of adult cells, such as occurs when blood cells fuse with hepatocytes during repair of damaged liver (Anderson *et al*. 2001; Jaenisch & Young 2008). In addition, it has been known for many years that adult urodele amphibians can regenerate limbs or the eye lens following injury; this involves dedifferentiation and subsequent transdifferentiation steps (Brockes & Kumar 2005).

The early studies involving somatic nuclear transfer indicated that adult cells can be reprogrammed to pluripotency. However, the mechanistic and practical applications of inducing pluripotency in adult cells have only become apparent in the last 2 or 3 years, with the emergence of a new research area: induced pluripotent stem cells (iPS cells). The original report demonstrated that retrovirus-mediated transduction of mouse fibroblasts with four transcription factors (Oct-3/4, Sox2, KLF4 and c-Myc; figure 1) that are highly expressed in ES cells could induce the fibroblasts to become pluripotent (Takahashi & Yamanaka 2006). Since then, rapid progress has been made: iPS cells can be generated from adult human cells (Takahashi *et al*. 2007; Yu *et al*. 2007; Park *et al*. 2008*a*); cells from a range of tissues can be reprogrammed (Aasen *et al*. 2008; Aoi *et al*. 2008); and iPS cells can be generated from patients with specific diseases (Dimos *et al*. 2008; Park *et al*. 2008*b*). The number of transcription factors required to generate iPS cells has been reduced (Kim *et al*. 2008); the efficiency of iPS cell generation increased (Wernig *et al*. 2007); and techniques devised that obviate the need for retroviral vectors (Okita *et al*. 2008; Stadtfeld *et al*. 2008). These latter developments are very important for future clinical applications, since the early mice generated from iPS cells developed tumours at high frequency (Takahashi & Yamanaka 2006; Yamanaka 2007). Without a doubt, this is currently the most exciting and rapidly moving area of stem cell research.

## 2. Current clinical applications of stem cells

In all the publicity that surrounds embryonic and iPS cells, people tend to forget that stem cell-based therapies are already in clinical use and have been for decades. It is instructive to think about these treatments, because they provide important caveats about the journey from proof-of-principle in the laboratory to real patient benefit in the clinic. These caveats include efficacy, patient safety, government legislation and the costs and potential profits involved in patient treatment.

Haemopoietic stem cell transplantation is the oldest stem cell therapy and is the treatment that is most widely available (Perry & Linch 1996; Austin *et al*. 2008). The stem cells come from bone marrow, peripheral blood or cord blood. For some applications, the patient's own cells are engrafted. However, allogeneic stem cell transplantation is now a common procedure for the treatment of bone marrow failure and haematological malignancies, such as leukaemia. Donor stem cells are used to reconstitute immune function in such patients following radiation and/or chemotherapy. In the UK, the regulatory framework put in place for bone marrow transplantation has now an extended remit, covering the use of other tissues and organs (Austin *et al*. 2008).

Advances in immunology research greatly increased the utility of bone marrow transplantation, allowing allograft donors to be screened for the best match in order to prevent rejection and graft-versus-host disease (Perry & Linch 1996). It is worth remembering that organ transplantation programmes have also depended on an understanding of immune rejection, and drugs are available to provide effective long-term immunosuppression for recipients of donor organs. Thus, while it is obviously desirable for new stem cell treatments to involve the patient's own cells, it is certainly not essential.

Two major advantages of haemopoietic stem cell therapy are that there is no need to expand the cells in culture or to reconstitute a multicellular tissue architecture prior to transplantation. These hurdles have been overcome to generate cultured epidermis to provide autologous grafts for patients with full-thickness wounds, such as third-degree burns. Proof-of-principle was established in the mid-1970s, with clinical and commercial applications following rapidly (Green 2008). Using a similar approach, limbal stem cells have been used successfully to restore vision in patients suffering from chemical destruction of the cornea (De Luca *et al*. 2006).

*Ex vivo* expansion of human epidermal and corneal stem cells frequently involves culture on a feeder layer of mouse fibroblastic cells in medium containing bovine serum. While it would obviously be preferable to avoid animal products, there has been no evidence over the past 30 years that exposure to them has had adverse effects on patients receiving the grafts. The ongoing challenges posed by epithelial stem cell treatments include improved functionality of the graft (e.g. through generation of epidermal hair follicles) and improved surfaces on which to culture the cells and apply them to the patients. The need to optimize stem cell delivery is leading to close interactions between the stem cell community and bioengineers. In a recent example, a patient's trachea was repaired by transplanting a new tissue constructed in culture from donor decellularized trachea seeded with the patient's own bone marrow cells that had been differentiated into cartilage cells (Macchiarini *et al*. 2008).

Whereas haemopoietic stem cell therapies are widely available, treatments involving cultured epidermis and cornea are not. In countries where cultured epithelial grafts are available, the number of potential patients is relatively small and the treatment costly. Commercial organizations that sell cultured epidermis for grafting have found that it is not particularly profitable, while in countries with publicly funded healthcare the need to set up a dedicated laboratory to generate the grafts tends to make the financial cost–benefit ratio too high (Green 2008).

Clinical studies over the last 10 years suggest that stem cell transplantation also has potential as a therapy for neurodegenerative diseases. Clinical trials have involved grafting brain tissue from aborted foetuses into patients with Parkinson's disease and Huntington's disease (Dunnett *et al*. 2001; Wright & Barker 2007). While some successes have been noted, the outcomes have not been uniform and further clinical trials will involve more refined patient selection, in an attempt to predict who will benefit and who will not. Obviously, aside from the opposition in many quarters to using foetal material, there are practical challenges associated with availability and uniformity of the grafted cells and so therapies with pure populations of stem cells are an important, and achievable (Conti *et al*. 2005; Lowell *et al*. 2006), goal.

No consideration of currently available stem cell therapies is complete without reference to gene therapy. Here, there have been some major achievements, including the successful treatment of children with X-linked severe combined immunodeficiency. However, the entire gene therapy field stalled when several of the children developed leukaemia as a result of integration of the therapeutic retroviral vector close to the LMO2 oncogene locus (Gaspar & Thrasher 2005; Pike-Overzet *et al*. 2007). Clinical trials have since restarted, and in an interesting example of combined gene/stem cell therapy, a patient with an epidermal blistering disorder received an autologous graft of cultured epidermis in which the defective gene had been corrected *ex vivo* (Mavilio *et al*. 2006).

These are just some examples of treatments involving stem cells that are already in the clinic. They show how the field of stem cell transplantation is interlinked with the fields of gene therapy and bioengineering, and how it has benefited from progress in other fields, such as immunology. Stem cells undoubtedly offer tremendous potential to treat many human diseases and to repair tissue damage resulting from injury or ageing. The danger, of course, lies in the potentially lethal cocktail of desperate patients, enthusiastic scientists, ambitious clinicians and commercial pressures (Lau *et al*. 2008). Internationally agreed, and enforced, regulations are essential in order to protect patients from the dangers of stem cell tourism, whereby treatments that have not been approved in one country are freely available in another (Hyun *et al*. 2008).

## 3. What are the big questions in the field?

Three questions in stem cell research are being hotly pursued at present. What are the core genetic and epigenetic regulators of stem cells? What are the extrinsic, environmental factors that influence stem cell renewal and differentiation? And how can the answers to the first two questions be harnessed for clinical benefit?

## 4. Core genetic and epigenetic regulators

Considerable progress has already been made in defining the transcriptional circuitry and epigenetic modifications associated with pluripotency (Jaenisch & Young 2008). This research area is moving very rapidly as a result of tremendous advances in DNA sequencing technology, bioinformatics and computational biology. Chromatin immunoprecipitation combined with microarray hybridization or DNA sequencing (Mathur *et al*. 2008) is being used to identify transcription factor-binding sites, and bioinformatics techniques have been developed to allow integration of data obtained by the different approaches. It is clear that pluripotency is also subject to complex epigenetic regulation, and high throughput genome-scale DNA methylation profiling has been developed for epigenetic profiling of ES cells and other cell types (Meissner *et al*. 2008).

Oct4, Nanog and Sox2 are core transcription factors that maintain pluripotency of ES cells. These factors bind to their own promoters, forming an autoregulatory loop. They occupy overlapping sets of target genes, one set being actively expressed and the other, comprising genes that positively regulate lineage selection, being actively silenced (Jaenisch & Young 2008; Mathur *et al*. 2008; Silva & Smith 2008). Nanog stabilizes pluripotency by limiting the frequency with which cells commit to differentiation (Chambers *et al*. 2007; Torres & Watt 2008). The core pluripotency transcription factors also regulate, again positively and negatively, the microRNAs that are involved in controlling ES cell self-renewal and differentiation (Marson *et al*. 2008).

As the basic mechanisms that maintain the pluripotent state of ES cells are delineated, there is considerable interest in understanding how pluripotency is re-established in adult stem cells. It appears that some cell types are more readily reprogrammed to iPS cells than others (Aasen *et al*. 2008; Aoi *et al*. 2008), and it is interesting to speculate that this reflects differences in endogenous expression of the genes required for reprogramming or in responsiveness to overexpression of those genes (Hochedlinger *et al*. 2005; Markoulaki *et al*. 2009). Another emerging area of investigation is the relationship between the epigenome of pluripotent stem cells and cancer cells (Meissner *et al*. 2008).

Initial attempts at defining ‘stemness’ by comparing the transcriptional profiles of ES cells, neural and haemopoietic stem cells (Ivanova *et al*. 2002; Ramalho-Santos *et al*. 2002) have paved the way for more refined comparisons. For example, by comparing the gene expression profiles of adult neural stem cells, ES-derived and iPS-derived neural stem cells and brain tumour stem cells, it should be possible both to validate the use of ES-derived stem cells for brain repair and to establish the cell of origin of brain tumour initiating cells. Furthermore, it is anticipated that new therapeutic targets will be identified from molecular profiling studies of different stem cell populations.

As gene expression profiling becomes more sophisticated, the question of ‘what is a stem cell?’ can be addressed in new ways. Several studies have used single cell expression microarrays to identify new stem cell markers (Jensen & Watt 2006). Stem cells are well known to exhibit different proliferative and differentiation properties in culture, during tissue injury and in normal tissue homeostasis, raising the question of which elements of the stem cell phenotype are hard-wired versus a response to environmental conditions.

**Figure 3.**
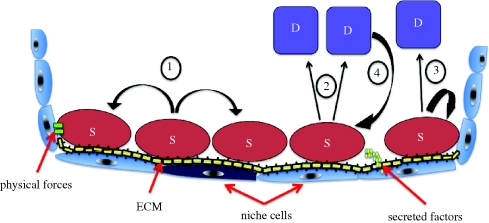
The stem cell niche. Stem cells (S) are shown dividing symmetrically to produce two stem cells (1) or two differentiated cells (D) (2), or undergoing asymmetric division to produce one stem cell and one differentiated cell (3). Under some circumstances, a differentiated cell can re-enter the niche and become a stem cell (4). Different components of the stem cell niche are illustrated: extracellular matrix (ECM), cells in close proximity to stem cells (niche cells), secreted factors (such as growth factors) and physical factors (such as oxygen tension, stiffness and stretch).

One of the growing trends in stem cell research is the contribution of mathematical modelling. This is illustrated in the concept of transcriptional noise: the hypothesis that intercellular variability is a manifestation of ‘noise’ in gene expression levels, rather than stable phenotypic variation (Chang *et al*. 2008). Studies with clonal populations of haemopoietic progenitor cells have shown that slow fluctuations in protein levels can produce cellular heterogeneity that is sufficient to affect whether a given cell will differentiate along the myeloid or erythroid lineage (Chang *et al*. 2008). Mathematical approaches are also used increasingly to model observed differences in cell behaviour *in vivo*. In studies of adult mouse interfollicular epidermis, it is observed that cells can divide to produce two undifferentiated cells, two differentiated cells or one of each (figure 3); it turns out that this can be explained in terms of the stochastic behaviour of a single population of cells rather than by invoking the existence of discrete types of stem and progenitor cell (Clayton *et al*. 2007).

## 5. Extrinsic regulators

There is strong evidence that the behaviour of stem cells is strongly affected by their local environment or niche (figure 3). Some aspects of the stem cell environment that are known to influence self-renewal and stem cell fate are adhesion to extracellular matrix proteins, direct contact with neighbouring cells, exposure to secreted factors and physical factors, such as oxygen tension and sheer stress (Watt & Hogan 2000; Morrison & Spradling 2008). It is important to identify the environmental signals that control stem cell expansion and differentiation in order to harness those signals to optimize delivery of stem cell therapies.

Considerable progress has been made in directing ES cells to differentiate along specific lineages *in vitro* (Conti *et al*. 2005; Lowell *et al*. 2006; Izumi *et al*. 2007) and there are many *in vitro* and murine models of lineage selection by adult tissue stem cells (e.g. Watt & Collins 2008). It is clear that in many contexts the Erk and Akt pathways are key regulators of cell proliferation and survival, while pathways that were originally defined through their effects in embryonic development, such as Wnt, Notch and Shh, are reused in adult tissues to influence stem cell renewal and lineage selection. Furthermore, these core pathways are frequently deregulated in cancer (Reya *et al*. 2001; Watt & Collins 2008). In investigating how differentiation is controlled, it is not only the signalling pathways themselves that need to be considered, but also the timing, level and duration of a particular signal, as these variables profoundly influence cellular responses (Silva-Vargas *et al*. 2005). A further issue is the extent to which directed ES cell differentiation *in vitro* recapitulates the events that occur during normal embryogenesis and whether this affects the functionality of the differentiated cells (Izumi *et al*. 2007).

For a more complete definition of the stem cell niche, researchers are taking two opposite and complementary approaches: recreating the niche *in vitro* at the single cell level and observing stem cells *in vivo. In vivo* tracking of cells is possible because of advances in high-resolution confocal microscopy and two-photon imaging, which have greatly increased the sensitivity of detecting cells and the depth of the tissue at which they can be observed. Studies of green fluorescent protein-labelled haemopoietic stem cells have shown that their relationship with the bone marrow niche, comprising blood vessels, osteoblasts and the inner bone surface, differs in normal, irradiated and c-Kit-receptor-deficient mice (Lo Celso *et al*. 2009; Xie *et al*. 2009). In a different approach, *in vivo* bioluminescence imaging of luciferase-tagged muscle stem cells has been used to reveal their role in muscle repair in a way that is impossible when relying on retrospective analysis of fixed tissue (Sacco *et al*. 2008).

The advantage of recreating the stem cell niche *in vitro* is that it is possible to precisely control individual aspects of the niche and measure responses at the single cell level. Artificial niches are constructed by plating cells on micropatterned surfaces or capturing them in three-dimensional hydrogel matrices. In this way, parameters such as cell spreading and substrate mechanics can be precisely controlled (Watt *et al*. 1988; Théry *et al*. 2005; Chen 2008). Cells can be exposed to specific combinations of soluble factors or to tethered recombinant adhesive proteins. Cell behaviour can be monitored in real time by time-lapse microscopy, and activation of specific signalling pathways can be viewed using fluorescence resonance energy transfer probes and fluorescent reporters of transcriptional activity. It is also possible to recover cells from the *in vitro* environment, transplant them *in vivo* and monitor their subsequent behaviour. One of the exciting aspects of the reductionist approach to studying the niche is that it is highly interdisciplinary, bringing together stem cell researchers and bioengineers, and also offering opportunities for interactions with chemists, physicists and materials scientists.

## 6. Future clinical applications of stem cell research

Almost every day there are reports in the media of new stem cell therapies. There is no doubt that stem cells have the potential to treat many human afflictions, including ageing, cancer, diabetes, blindness and neurodegeneration. Nevertheless, it is essential to be realistic about the time and steps required to take new therapies into the clinic: it is exciting to be able to induce ES cells to differentiate into cardiomyocytes in a culture dish, but that is only one very small step towards effecting cardiac repair. The overriding concerns for any new treatment are the same: efficacy, safety and affordability.

In January 2009, the US Food and Drug Administration approved the first clinical trial involving human ES cells, just over 10 years after they were first isolated. In this trial, the safety of ES cell-derived oligodendrocytes in repair of spinal cord injury will be evaluated (http://www.geron.com). There are a large number of human ES cell lines now in existence and banking of clinical grade cells is underway, offering the opportunity for optimal immunological matching of donors and recipients. Nevertheless, one of the attractions of transplanting iPS cells is that the patient's own cells can be used, obviating the need for immunosuppression. Discovering how the pluripotent state can be efficiently and stably induced and maintained by treating cells with pharmacologically active compounds rather than by genetic manipulation is an important goal (Silva *et al*. 2008).

An alternative strategy to stem cell transplantation is to stimulate a patient's endogenous stem cells to divide or differentiate, as happens naturally during skin wound healing. It has recently been shown that pancreatic exocrine cells in adult mice can be reprogrammed to become functional, insulin-producing beta cells by expression of transcription factors that regulate pancreatic development (Zhou *et al*. 2008). The idea of repairing tissue through a process of cellular reprogramming *in situ* is an attractive paradigm to be explored further.

A range of biomaterials are already in clinical use for tissue repair, in particular to repair defects in cartilage and bone (Kamitakahara *et al*. 2008). These can be considered as practical applications of our knowledge of the stem cell microenvironment. Advances in tissue engineering and materials science offer new opportunities to manipulate the stem niche and either facilitate expansion/differentiation of endogenous stem cells or deliver exogenous cells. Resorbable scaffolds can be exploited for controlled delivery and release of small molecules, growth factors and peptides. Conversely, scaffolds can be designed that are able to capture unwanted tissue debris that might impede repair. Hydrogels that can undergo controlled sol–gel transitions could be used to release stem cells once they have integrated within the target tissue.

Although most of the new clinical applications of stem cells have a long lead time, applications of stem cells in drug discovery are available immediately. Adult tissue stem cells, ES cells and iPS cells can all be used to screen for compounds that stimulate self-renewal or promote specific differentiation programmes. Finding drugs that selectively target cancer stem cells offers the potential to develop cancer treatments that are not only more effective, but also cause less collateral damage to the patient's normal tissues than drugs currently in use. In addition, patient-specific iPS cells provide a new tool to identify underlying disease mechanisms. Thus stem cell-based assays are already enhancing drug discovery efforts.

## 7. Conclusion

Amid all the hype surrounding stem cells, there are strong grounds for believing that over the next 50 years our understanding of stem cells will revolutionize medicine. One of the most exciting aspects of working in the stem cell field is that it is truly multidisciplinary and translational. It brings together biologists, clinicians and researchers across the physical sciences and mathematics, and it fosters partnerships between academics and the biotech and pharmaceutical industries. In contrast to the golden era of developmental biology, one of stem cell research's defining characteristics is the motivation to benefit human health.
